# Novel insights into the METTL3-METTL14 complex in musculoskeletal diseases

**DOI:** 10.1038/s41420-023-01435-9

**Published:** 2023-05-18

**Authors:** Yeqiu Xu, Yuanzhuang Zhang, Yinzhou Luo, Guanzhen Qiu, Jie Lu, Ming He, Yong Wang

**Affiliations:** 1 Fourth Department of Orthopedic Surgery, Central Hospital Affiliated to Shenyang Medical College, 110024 Shenyang, Liaoning People’s Republic of China; 2 Department of Cardiology, Shenyang Fourth People’s Hospital, China Medical University, 110031 Shenyang, Liaoning People’s Republic of China; 3 Department of Orthopedics, Shengjing Hospital of China Medical University, 110004 Shenyang, Liaoning People’s Republic of China

**Keywords:** Diseases, Epigenetics

## Abstract

N6-methyladenosine (m^6^A) modification, catalyzed by methyltransferase complexes (MTCs), plays many roles in multifaceted biological activities. As the most important subunit of MTCs, the METTL3-METTL14 complex is reported to be the initial factor that catalyzes the methylation of adenosines. Recently, accumulating evidence has indicated that the METTL3-METTL14 complex plays a key role in musculoskeletal diseases in an m^6^A-dependent or -independent manner. Although the functions of m^6^A modifications in a variety of musculoskeletal diseases have been widely recognized, the critical role of the METTL3-METTL14 complex in certain musculoskeletal disorders, such as osteoporosis, osteoarthritis, rheumatoid arthritis and osteosarcoma, has not been systematically revealed. In the current review, the structure, mechanisms and functions of the METTL3-METTL14 complex and the mechanisms and functions of its downstream pathways in the aforementioned musculoskeletal diseases are categorized and summarized.

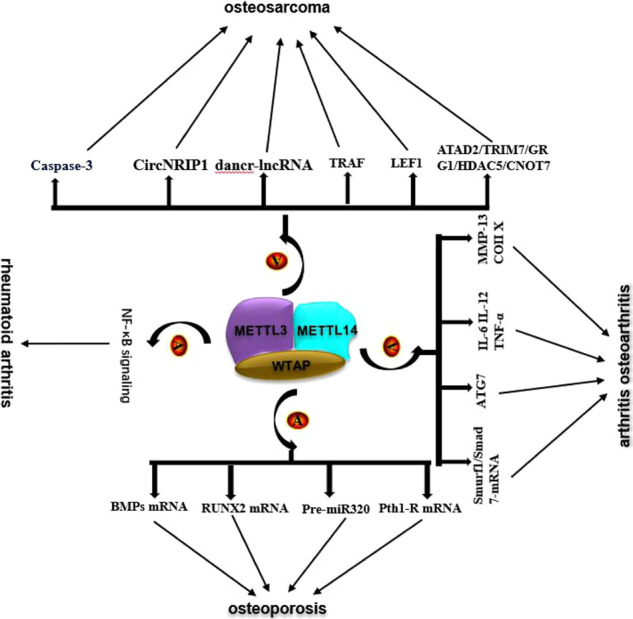

## Facts

1. The molecular mechanisms of certain musculoskeletal diseases, such as arthritis osteoarthritis (OA), osteoporosis (OP), osteosarcoma (OS), and rheumatoid arthritis (RA), are intricate and ambiguous.
2. As a reversible epigenetic regulator, N6-methyladenosine (m^6^A) modification catalyzed by methyltransferase complexes (MTCs) is implicated in numerous human disorders, including musculoskeletal diseases.
3. The METTL3-METTL14 complex participates in aspects of RNA metabolism, such as RNA alternative splicing, transport, and stability and microRNA maturation and decay.
4. The structure of the METTL3-METTL14 complex allows it to exert its catalytic function during m^6^A modification.

## Open questions

1. What is the structure of the METTL3-METTL14 complex?
2. What are the functional roles and corresponding regulatory mechanisms of the METTL3-METTL14 complex in musculoskeletal diseases?
3. What clinical applications related to the METTL3-METTL14 complex exist?

## Introduction

The musculoskeletal system, also named the motor system, is mainly composed of bones and skeletal muscles and is directly involved in the motor function of the human body [1]. Musculoskeletal disorders are increasingly recognized as a leading cause of stress and disability in working-age adults [2]. There are more than 100 types of musculoskeletal diseases, including arthritis osteoarthritis (OA), osteoporosis (OP), osteosarcoma (OS), and rheumatoid arthritis (RA). The pathogenesis of these disorders is intricate and is far from being fully understood [3–5]. It is generally believed that the occurrence of these musculoskeletal diseases is closely related to multiple factors, such as environmental, organic, hereditary, and epigenetic factors, including m^6^A modification [5–11].

Recently, increasing attention has been given to the role of epigenetic mechanisms, including heritable changes in gene function without gene sequence alteration, in musculoskeletal diseases [12, 13]. Previous studies have suggested that noncoding RNAs such as microRNAs are the main sites of RNA epigenetic modification [14]. New research has found that RNA m^6^A modification is widely distributed in organisms and plays a dynamic regulatory role [15]. Many studies have demonstrated that m^6^A modification is involved in the occurrence and development of musculoskeletal diseases [16, 17].

m^6^A, first discovered in the L cells of mammalian mice in the 1970s, is the dynamic reversible chemical modification of the N6 site of adenosine in specific RNA sequences [18, 19]. m^6^A is an internal biological marker, the deposition of which is mainly catalyzed by methyltransferases called “writers”; demethylation occurs under the catalysis of demethylases called “erasers”; and marks are identified by a series of specific RNA binding proteins called “readers”. As initiating factors, m^6^A writers include several factors with methylation ability, including the METTL3-METTL14 complex, METTL5, METTL16, ZCCHC4, and certain accessory components, such as WTAP, RBM15 and KIAA1429 [20–24]. Among them, the METTL3-METTL14 complex is the most interesting from a research perspective [25–28]. METTL3 and METTL14 closely associate with each other and form a stable heterodimer at a ratio of 1:1. The METTL3-METTL14 complex heterodimer has improved structural stability and catalytic activity [29–32]. With increased research into m^6^A modification, the biological functions of the METTL3-METTL14 complex in musculoskeletal diseases have been extensively studied [33]. This review introduces the main features of the METTL3-METTL14 complex and its working mechanism in m^6^A modification and summarizes recent progress in understanding the role of the METTL3-METTL14 complex in certain musculoskeletal diseases, such as OP, OS, RA and OA. Additionally, this review provides an overview of the regulatory mechanisms of the METTL3-METTL14 complex in the abovementioned disorders.

## Structure and working mechanism of the METTL3-METTL14 complex

As the most important factor of methyltransferases, the METTL3-METTL14 complex was first purified from HeLa cell nuclear extracts in 1994 [34]. The METTL3-METTL14 complex, the core of which comprises METTL3 and METTL14, includes three interacting but independent components that are separable under invariant conditions [35]. The stable heterodimer automatically formed by METTL3-METTL14 is the structural basis that underlies the ability of methyltransferases to achieve catalytic functions such as binding to target RNA and transferring methyl groups.

### The structure of METTL3

**Fig. 1:**
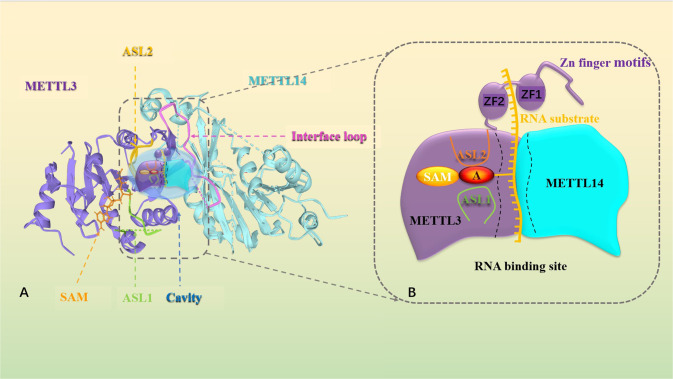
Schematic diagram of METTL3-METTL14 complex structure. **A** Annotated model of the METTL3-METTL14 complex structure. The METTL3 (purple) and METTL14 (blue) complex structure is shown. SAM (orange sticks) binds one side of the catalytic cavity. The concave space between the MTDs is surrounded by the ASL1 loop (green), ASL2 loop (yellow), and interface loop (pink). **B** Proposed model for RNA methylation by the METTL3-METTL14 complex. The substrate RNA binds to the active center composed of the METTL3-METTL4 heterodimer, and the receptor adenine is localized in the METTL3 catalytic pocket between the ASL1 and ASL2 loops. METTL3 catalyzes the transfer of methyl groups from SAM to the acceptor adenine. ZFD in the N-terminal region of METTL3 facilitates substrate RNA recognition.

METTL3, which is 580 amino acids in length, is primarily composed of a zinc finger domain (ZFD), which contains two tandem CCCH-type zinc-binding motifs connected by antiparallel β-sheets, and a methyltransferase domain (MTD). As shown in Fig. 1A, the MTD of METTL3, also named MTD3 (residues 357–580) is a highly conserved open hollow cavity that is the most significant catalytic site, and MTD3 is responsible for binding to the donor substrate through various types of chemical bonds [32, 36]. A primary-sequence level conserved S-adenosylmethionine (SAM) binding site that can bind to SAM via hydrogen bonds exists on one side of the cavity. The rest of the cavity is presumably for binding of RNA substrates [32]. By using electron scanning, Wang and colleagues generated clear images of the METTL3 hollow cavity combined with SAM. The catalytic cavity is also surrounded by three main loops: two of these loops (active site loop 1 (ASL1), which is a partially disordered loop containing Asp395-Thr408, and active site loop2 (ASL2), a fully ordered loop containing Arg508-Lys513) surround the SAM-binding site. The third loop has a larger interface that extensively contacts METTL14. This loop provides the structural basis for proper folding and stability of the heterodimer [32, 37, 38] (Fig. 1A).

### The structure of METTL14

METTL14 is located on chromosome 4q26 and contains 12 exons. METTL14, 456 amino acids in length, is a homolog of METTL3 with 43% homology [29, 39]. Previous studies have revealed that both METTL3 and METTL14 have homologous methyltransferase domains [40]. However, unlike MTD3, the catalytic cavity of the MTD in METTL14 (named MTD14, residues 111–456) is low. Due to the lack of residues that form hydrogen bonds with the ribose hydroxyls of SAM, METTL14 cannot bind SAM [37, 40, 41]. Similarly, it has been reported that METTL14 is catalytically inactive in the METTL3-METTL14 complex because it contains a degenerate active site that is unable to accommodate donor and acceptor substrates [37]. This finding indicates that METTL14 contains a nonfunctional catalytic site. Additionally, METTL14 does not have a ZFD, which is responsible for the recognition and binding of single-stranded RNAs containing the GGACU motif [31]. However, MTD14, which is similar to methyltransferases of the target recognition domain (TRD) of class I DNA n6-adenine methyltransferases, acts as a substrate-binding scaffold to enhance the methyltransferase activity of METTL3 [39]. The two ends of METTL14, the N-terminus and the C-terminus, have helical extensions that are parallel to each other, pass through one face of MTD14, and make extensive contact with MTD3 through the interface loop and shorter helical segments of MTD3 [42] (Fig. 1A).

### Mechanisms of the METTL3-METTL14 complex

As shown in Fig. 1B, MTD3 and MTD14, two methyltransferase domains of the heterodimeric complex, maintain their conformation via various hydrophobic and polar contacts. The hollow cavity between MTD3 and MTD14 that forms the active site is important for binding substrate RNA [32, 43]. ZFD, which is flexible and partially folded with MTD3, recognizes single-stranded RNAs containing a 5’-GGACU-3’ consensus sequence, and the auxiliary active region completes the binding to substrate RNAs [31]. On one side of the MTD3 cavity exists a site that accommodates a methyl donor (SAM or SAH), which cooperates with the active region to transfer the methyl group to the adenosine of the specific RNA motif. The important active sites of METTL14 (R245, R249, R254, R255, K297, and R298) are involved in the formation of a unique conformation at the contact site of the heterodimer and are responsible for enhancing the RNA binding ability [39]. In addition, METTL14 can directly bind to the RNA substrate by recognizing the basic patch, which determines the specificity of RNA sequence recognition by the heterodimer [32].

As the core methyltransferase subunit, the METTL3-METTL14 complex affects various aspects of RNA metabolism, such as RNA alternative splicing, transport, and stability and microRNA maturation and decay. Alternative splicing, by which the exons of primary transcripts from genes can be combined in different arrangements, is primarily responsible for the substantial cellular complexity [44]. METTL3 affected the osteogenic differentiation ability of bone marrow mesenchymal stem cells (BMSCs). Knockdown of METTL3 in BMSCs decreased the mRNA expression of two splice variants of vascular endothelial growth factor A (VEGFA), vegfa-164 and vegfa-188, but did not affect the expression of vegfa-120 [45]. A recent report showed that METTL14 is a critical regulator of the maturation of oligodendrocyte lineage cells. Ablation of METTL14 led to aberrant splicing of numerous RNA transcripts in oligodendrocyte lineage cells [46].

Efficient transport of messenger RNA from the nucleus to the cytoplasmic sites of active translation is a fundamental feature of eukaryotes [47]. By an RT‒qPCR-based nucleocytoplasmic fractionation assay, Li and colleagues found that knockdown of METTL3 facilitated the nuclear retention of TNF receptor-associated factor 6 (TRAF6) transcripts but decreased the cytoplasmic abundance of TRAF6 mRNA during osteoclast differentiation [33].

Notably, METTL3-METTL14 complex-methylated mRNAs are preferentially recognized by certain “readers”, such as YTHDF members, which triggers their rapid degradation [19, 48]. The METTL3-METTL14 complex is also involved in the regulation of the fates of noncoding RNAs. METTL3 and METTL14 participate in the maturation of primary (pri-)miRNAs such as miR-25-3p, pri-miR221/222, pri-miR-1246, pri-miR-375 and pri-miR-126 in different cancers [49–53]. Additionally, METTL3 and METTL14 control the expression and stability of several long noncoding RNAs, such as LINC00958, MALAT1, LINC00942 and XIST [54–57].

Interestingly, in addition to RNA metabolism, it was recently reported that METTL3 participates in the regulation of chromatin state and transcription. METTL3, in collaboration with YTHDC1, decreased the stability of chromosome-associated regulatory RNAs (carRNAs) through NEXT-mediated nuclear degradation and ultimately changed the chromatin state and transcription [58]. Xu and colleagues found that METTL3 was essential for the integrity of intracisternal A particle (IAP) heterochromatin in mouse embryonic stem cells. Knockout of METTL3 attenuated the abundance of various heterochromatin marks of METTL3-targeted IAPs while upregulating IAP transcription in an m^6^A-dependent manner [59].

## Roles of the METTL3-METTL14 complex in musculoskeletal diseases

As prevalent disorders among populations with frequent comorbidities, musculoskeletal diseases are responsible for a significant portion of the global disease burden [60, 61]. The METTL3-METTL14 complex is extensively involved in various diseases, such as cancers, cardiovascular disorders, neurological disorders and musculoskeletal diseases [36, 42, 62–64]. In the following section, we will discuss recent studies of the role of the METTL3-METTL14 complex in certain musculoskeletal diseases, such as OP, OA, RA and OS.

### The roles of METTL3 and METTL14 in osteoporosis

OP is a systemic bone disease characterized by decreased bone mass and microarchitectural deterioration, leading to increased bone fragility and susceptibility to fractures [65]. Recent evidence has revealed that the METTL3-METTL14 complex regulates OP by affecting multiple signaling axes through m^6^A modification [45, 66–68].

**Fig. 2:**
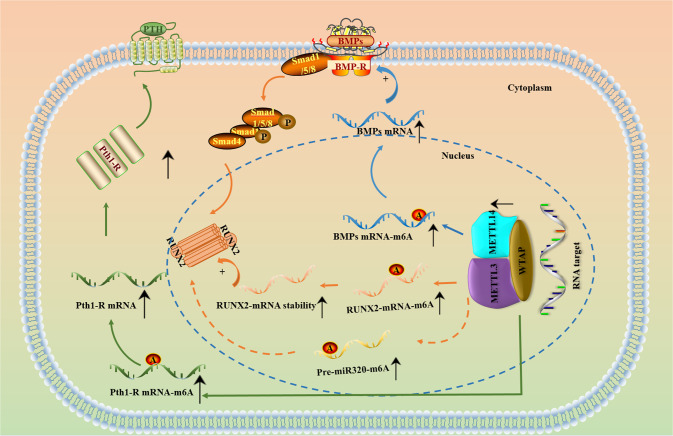
Several signaling pathways regulated by METTL3 and METTL14 in OP. METTL3 and METTL14 regulate mRNA expression in an m^6^A-dependent manner and thus regulate OP processes through several signaling pathways. A:m^6^A; P:phosphorylation.

It was discovered that the expression of METTL3 and METTL14 and the overall m^6^A level were decreased in the bone tissues of three patients with osteoporosis [69]. Upregulation of METTL3 promotes bone formation in mice and facilitates osteogenic differentiation of BMSCs via induction of m^6^A methylation and the expression of several osteogenesis-associated genes, such as RUNX family transcription factor 2 (RUNX2), aleurain-like protease (ALP) and bone gamma-carboxyglutamate protein (BGLAP) [69]. RUNX2 is a transcription factor that plays a vital role in the differentiation of osteoblasts, enhances bone mineralization, and participates in various processes in OP [69, 70]. Similarly, another study claimed that METTL3 knockdown decreased the m^6^A modification level of BMP mRNA, inhibited the bone morphogenetic protein (BMP)/SMAD pathway, and induced pathological features of OP [71]. The above findings suggest that the role of METTL3 in OP is mainly achieved by its catalysis of m^6^A modification of various mRNAs. Another downstream pathway of METTL3 in OP is the parathyroid hormone (PTH)/parathyroid hormone 1 receptor (PTH1R) signaling pathway. Wu and coworkers found that knockout of METTL3 reduced the translation efficiency of PTH1R mRNA and disrupted PTH-induced osteogenic and adipogenic responses in MSCs in an m^6^A manner [72] (Fig. 2, Table 1).

**Table 1:** Summary of included studies about the regulatory role of METTL3-14 complex in musculoskeletal diseases.

Diseases	Component	Modus	Pathway/target	Function	Regulation	Role in diseases	Ref
OP	METTL3	m6A-dependent	Pre-miR-320/RUNX2	Suppressor	Downregulation	Promotes bone formation	[69]
	METTL3	m6A-dependent	BMPs/Smads signaling	Suppressor	Downregulation	Promote osteogenic differentiation	[71]
	METTL3	m6A-dependent	PTH/PTH1R signaling	Suppressor	Downregulation	Decrease bone loss	[72]
	METTL14	m6A-dependent	DGCR8/miR-103-3p	Suppressor	Downregulation	Promoted osteoblast proliferation, differentiation, and matrix mineralization	[74]
OA	METTL3	m6A-dependent	ATG7/GATA4 signaling	Promotor	Upregulation	Promote the aging of FLSs	[77]
	METTL3	m6A-dependent	Smad/MAPK signaling	Suppressor	Downregulation	Inhibit the expression of osteoblast inflammatory factors	[78]
	METTL3	m6A-dependent	/	Promotor	Upregulation	Promote the degradation of extracellular matrix	[79]
RA	METTL3	m6A-dependent	NF-κB signaling	Promotor	Upregulation	Promotes LPS-mediated inflammatory responses in macrophages	[82]
	METTL3	m6A-dependent	NF-κB signaling	Promotor	Upregulation	Promotes activation and inflammation of FLSs	[83]
OS	METTL3	m6A-dependent	LEF1 mRNA	Oncogene	Upregulation	Promote the proliferation, migration and invasion of OS	[87]
	METTL3	m6A-dependent	TRAF6 mRNA	Oncogene	Upregulation	Regulation of epithelial-mesenchymal transition in OS cells	[9]
	METTL3	m6A-dependent	ATAD2 mRNA	Oncogene	Upregulation	Regulate OS proliferation, invasion and metastasis.	[88]
	METTL3	m6A-dependent	TRIM7 mRNA	Oncogene	Upregulation	Regulate OS proliferation, invasion and metastasis.	[89]
	METTL3	m6A-dependent	GRG1 mRNA	Oncogene	Upregulation	Regulate OS proliferation, invasion and metastasis.	[90]
	METTL3	m6A-dependent	HDAC5 mRNA	Oncogene	Upregulation	Regulate OS proliferation, invasion and metastasis.	[91]
	METTL3	m6A-dependent	CNOT7 mRNA	Oncogene	Upregulation	Regulate OS proliferation, invasion and metastasis.	[92]
	METTL3	m6A-dependent	DANCR lncRNA	Oncogene	Upregulation	Promote the proliferation, migration and invasion of OS	[93]
	METTL3	m6A-dependent	circNRIP1/miR-199a	Oncogene	Upregulation	Promote proliferation and migration, inhibit apoptosis	[94]
	METTL14	m6A-dependent	Caspase 3 Cleavage	Suppressor	Downregulation	Promote apoptosis of OS, and inhibit viability and proliferation of OS	[96]

In addition to the mechanisms mentioned above, METTL3 promotes the translation of a group of m^6^A-containing mRNAs, such as epidermal growth factor receptor (EGFR) and TAZ mRNAs, by interacting with the translation initiation machinery without the participation of m^6^A readers. Mechanistically, METTL3 enhances the translation of these mRNAs independently of the activity of methyltransferases or downstream m^6^A readers by recruiting eukaryotic translation initiation factor 3 (eIF3) to the translation initiation complex [73].

The biological function of METTL14 in OP has not been well explored. It has been reported that METTL14, as a downstream target of miR-103-3p, can be inhibited to further suppress the activity of osteoblasts. METTL14 can also regulate the processing of miR-103-3p by the DiGeorge syndrome critical region gene 8 (DGCR8) microprocessor complex subunit in an m^6^A-dependent manner, forming a negative feedback regulatory loop and promoting the proliferation, differentiation and matrix mineralization of osteoblasts. These studies also showed that METTL14 was positively associated with m^6^A modification and bone formation in older women and mice. After upregulation of METTL14 by vector transfection, m^6^A and bone formation indices were increased, but downregulation led to opposite results. This suggests that METTL14 plays an important role in OP in an m^6^A-dependent manner, but the specific mechanism needs further study [53, 74].

### The role of METTL3 in osteoarthritis

As a chronic degenerative joint condition, OA is one of the most common joint disorders in elderly individuals [75]. The pathological processes of OA involve disturbance of articular cartilage anabolism and catabolism, as well as degradation of the extracellular matrix [76]. Many epigenetic mechanisms are involved in the degeneration of articular cartilage during the pathogenesis of OA [7]. However, the role of m^6^A modification in OA has not been fully investigated.

An increased m^6^A modification level and upregulated METTL3 were found in fibroblast-like synoviocytes (FLSs) of patients with OA and in destabilization of the medial meniscus (DMM) mouse models. METTL3-mediated m^6^A modification attenuated the RNA stability of autophagy-related 7 (ATG7) in a YTHDF2-dependent manner and decreased the protein expression of ATG7, an E-1 enzyme crucial for the formation of autophagosomes, in OA FLSs. It was also observed that knockdown of METTL3 suppressed the expression of p16^INK4a^ and p21, the senescence-associated secretory phenotype (SASP) and the secretion of interleukin 1 beta (IL-1β), therefore alleviating the progression of OA in the DMM mouse model [77].

METTL3 has also been implicated in the inflammatory response in OA. METTL3 was downregulated in lipopolysaccharide (LPS)-treated preosteoblast MC3T3-E1 cells. METTL3 knockdown decreased the expression of osteogenic markers but regulated the expression of several proinflammatory cytokines, such as IL6, IL12, and TNF-α, by increasing the phosphorylation of members of the MAPK signaling pathway. METTL3 knockdown promoted the expression and stability of certain negative regulators of Smad signaling, such as Smad7 and SMAD-specific E3 ubiquitin protein ligase 1 (Smurf1) [78].

**Fig. 3:**
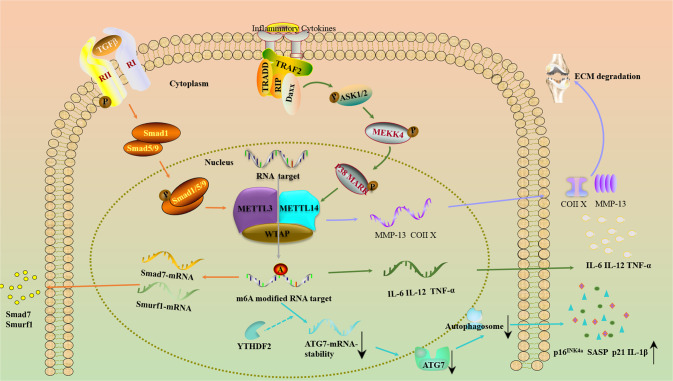
Several signaling pathways regulated by METTL3 in OA. METTL3 methylates target transcripts, resulting in m^6^A marks that regulate RNA expression. TGF-β, transforming growth factor-β.

From another perspective, METTL3 controls degradation of the extracellular matrix (ECM) during the progression of OA. Inconsistent with the findings above, Liu and associates found that METTL3 was increased in interleukin 1 beta (IL-1β)-treated ATDC5 cells. Silencing of METTL3 via transfection of METTL3 short hairpin RNA (shRNA) inhibited ECM degradation, as indicated by a reduction in matrix metallopeptidase 13 (MMP-13) and collagen type X (Coll X) but an increase in aggrecan and collagen type II (Coll II) [79] (Fig. 3, Table 1).

To date, there is limited research on METTL14 in OA.

### The role of METTL3 in rheumatoid arthritis

As the most common chronic inflammatory arthropathy, RA is characterized by persistent synovitis, systemic inflammation, and autoantibodies [80]. The immunopathogenesis of RA is intricate, and multiple factors, including environmental stress, the mucosal microbiome, genetic factors, and epigenetic marks, are involved in RA [81]. m^6^A modification was recently reported to participate in RA [16].

METTL3 was upregulated in patients with RA and in LPS-induced pTHP-1 macrophages, and elevated METTL3 was closely associated with the C-reactive protein (CRP) level and erythrocyte sedimentation rate (ESR), two markers of RA disease activity. Functionally, it was demonstrated that upregulation of METTL3 suppressed the inflammatory response of pTHP-1 macrophages by modulating the nuclear translocation of phosphorylated nuclear factor kappa B (NF-κB), a classic transcription factor involved in inflammation [82] (Table 1).

Via functional METTL3-related in vitro and in vivo assays, Shi and colleagues found that knockdown of METTL3 suppressed the expression of several inflammatory factors, such as MMP-3, MMP-9, and interleukin (IL)-6, in human RA FLSs and in adjuvant-induced arthritis (AIA) rat models. Mechanistically, researchers have also shown that the function of METTL3 in RA is closely related to the activation of the NF-κB pathway [83]. Therefore, METTL3/NF-κB might be useful molecular targets in RA.

### The role of the METTL3-METTL14 complex in osteosarcoma

OS is the most common primary bone cancer in children and young adults and has a high mortality rate [84]. Due to its malignant biological behaviors, such as fast growth and early metastasis, the prognosis of OS is poor [85]. Recent evidence has indicated that m^6^A modification, the most common epigenetic modification, is extensively involved in multiple cancers and diseases, including OS [17, 86]. As the most important initiator of m^6^A, the METTL13-METTL14 complex participates in diverse pathological processes of OS via different mechanisms.

Generally, METTL3 regulates OS progression by modulating the stability of messenger RNAs (mRNAs). METTL3 was elevated in human OS tissues and cell lines. Mechanistically, METTL3 affected the stability of lymphoid enhancer-binding factor 1 (LEF1) mRNA and subsequently regulated the activity of the Wnt/β-catenin signaling pathway, ultimately controlling the malignant behaviors of OS cells, such as proliferation, migration and invasion [87]. A similar study indicated that METTL3 regulated OS cell epithelial-mesenchymal transition (EMT) by regulating the stability of TRAF6 mRNA [9]. In addition, it has been reported that METTL3 also regulates the stability of certain other mRNAs, such as ATPase family-containing AAA domain 2 (ATAD2), tripartite motif containing 7 (TRIM7), GTP-binding protein 1 (GRG1), histone deacetylase 5 (HDAC5) and CCR4-NOT transcription complex subunit 7 (CNOT7), in an m^6^A-dependent manner in OS [88–92].

In addition to mRNAs, recent evidence has indicated that METTL3 also determines the fate of long noncoding RNAs and circular RNAs, two major types of noncoding RNAs, in OS. METTL3 facilitates the m^6^A modification of differentiation antagonizing nonprotein coding RNA (DANCR), a well-known oncogenic lncRNA, increasing the stability of DANCR and therefore promoting the increases in OS cell proliferation, migration and invasion induced by DANCR [93]. In another study, Meng and colleagues revealed that circRNA nuclear receptor interacting protein 1 (circNRIP1) exerted oncogenic functions, such as promoting proliferation and migration and suppressing apoptosis, by altering Forkhead box C2 (FOXC2) expression by sponging microRNA-199a (miR-199a) in OS cells. Additionally, the researchers found that silencing METTL3 reduced the m^6^A modification and expression level of circNRIP1 [94].

**Fig. 4:**
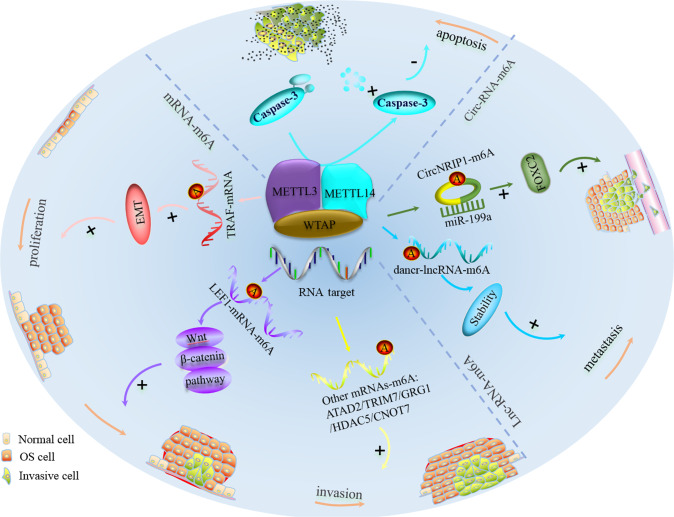
Several signalling pathways regulated by METTL3-METTL14 complex in OS. The METTL3-METTL14 complex affects OS progression through distinct RNA-mediated signaling pathways.

In contrast with the findings for METTL3, recent evidence has indicated that METTL14 is downregulated in OS [95, 96]. Functional cell proliferation, motility, and apoptosis assays showed that METTL14 acts as a tumor suppressor, inhibiting proliferation, migration, and invasion and inducing apoptosis through caspase 3 cleavage [96] (Fig. 4, Table 1).

## Conclusions and perspectives

An increasing number of studies have focused on the function of the METTL3-METTL14 complex in musculoskeletal diseases such as OP, OS, RA, and OA [97, 98]. The METTL3-METTL14 complex affects multiple cell signaling pathways, therefore regulating cell fate by altering the metabolism of RNAs in m^6^A-dependent and m^6^A-independent ways [73]. Specifically, as described earlier, RA and OA are inflammatory diseases, and in numerous studies, the METTL3-METTL14 complex has been shown to be involved in processes in RA and OA. The METTL3-METTL14 complex regulates the inflammatory response in RA and OA in an m^6^A-dependent manner through the Smad, MAPK and NF-κB signaling pathways [78, 79, 82]. However, the regulatory mechanisms in RA and OA are different. The METTL3-METTL14 complex controls the clearance of inflammatory mediators by regulating the ATG7 autophagosome and promotes the progression of OA by regulating the degradation of the extracellular matrix [77, 82]. The METTL3-METTL14 complex regulates the production of inflammatory mediators in RA [16].

Currently, there are limitations to the treatment of many musculoskeletal diseases, such as uncertain efficacy and harmful side effects [99–102]. Due to its dynamically reversible nature, the potential of interventions targeting METTL3-METTL14 complex-mediated m^6^A modification is substantial. Additionally, with the development of techniques to study RNA-modifying enzymes, such as high-throughput sequencing and self-assembled monolayer desorption/ionization (SAMDI) technology, the exploration of METTL3-METTL14-based targeted drugs may reveal promising strategies [103]. Recently, by high-throughput docking into the SAM-binding site and protein X-ray crystallography, Eliza and associates identified a small-molecule inhibitor of the METTL3-METTL14 complex named STM2457 [104]. STM2457 binds directly to the METTL3-METTL14 complex, reducing the m^6^A level and causing mRNA translation defects in human AML cell lines. The researchers also demonstrated that pharmacological inhibition of the METTL3-METTL14 complex in vivo extended survival in various mouse models of AML [104]. Collectively, these findings provide proof of concept for inhibition of the METTL3-METTL14 complex as a disease-specific therapeutic strategy. With the progress of relevant research, the mystery of the METTL3-METTL14 complex in musculoskeletal disorders will likely be revealed.

In addition to its role in musculoskeletal disorders, as the key human epitranscriptomic writer, the METTL3-METTL14 complex also exerts vital biological functions in various malignancies, including gastric cancer (GC), colorectal cancer (CRC), liver cancer (LC), and pancreatic cancer (PC) [105–108]. Therefore, a deep understanding of the molecular mechanisms of the METTL3-METTL14 complex in different diseases is of great significance for the early diagnosis and prognostication of patients. However, due to the complexity and diversity of the METTL3-METTL14 complex, applying the results of research of the METTL3-METTL14 complex in humans remains challenging. Although strategies related to the METTL3-METTL14 are promising, more research to explore the roles and detailed mechanisms of the METTL3-METTL14 complex in musculoskeletal disorders and other human diseases is needed.

## Data Availability

Not applicable.
