# Correction: Novel insights into the METTL3-METTL14 complex in musculoskeletal diseases

**DOI:** 10.1038/s41420-023-01480-4

**Published:** 2023-06-22

**Authors:** Yeqiu Xu, Yuanzhuang Zhang, Yinzhou Luo, Guanzhen Qiu, Jie Lu, Ming He, Yong Wang

**Affiliations:** 1 Fourth Department of Orthopedic Surgery, Central Hospital Affiliated to Shenyang Medical College, 110024 Shenyang, Liaoning People’s Republic of China; 2 Department of Cardiology, Shenyang Fourth People’s Hospital, China Medical University, 110031 Shenyang, Liaoning People’s Republic of China; 3 Department of Orthopedics, Shengjing Hospital of China Medical University, 110004 Shenyang, Liaoning People’s Republic of China

**Keywords:** Diseases, Epigenetics

Correction to: *Cell Death Discovery* (2023) **9**:170 10.1038/s41420-023-01435-9, published online 18 May 2023

In the published article the copyright holder is given erroneously. This should be corrected to “The Author(s)”.

The original article has been corrected.

